# Supplementary material for: The Systems Biology Research Tool: evolvable open-source software
Source: BMC Syst Biol. 2008 Jun 29;2:55. doi: 10.1186/1752-0509-2-55 (PMC2446383; doi:10.1186/1752-0509-2-55)
Supplement: Additional file 1 — SBRT Archive. An archive of the current version of the Systems Biology Research Tool. [file 1752-0509-2-55-S1.zip › sbrt-1.4.0/doc/developers_guide/api/sbrt/kernel/math/graph_theory/comp/PathFinder.html]

PathFinder


|  |  |  |
| --- | --- | --- |
| **Overview** \| **Package** \| **Class** \| **Use** \| **Tree** \| **Deprecated** \| **Index** \| **Help** | |  |
| PREV CLASS   NEXT CLASS | **FRAMES**    **NO FRAMES**     **All Classes** |  |
| SUMMARY: NESTED \| FIELD \| CONSTR \| METHOD | DETAIL: FIELD \| CONSTR \| METHOD |  |


---


## sbrt.kernel.math.graph\_theory.comp Class PathFinder<N>

```
java.lang.Object
  sbrt.kernel.math.graph_theory.comp.PathFinder<N>
```

**Type Parameters:**: `N` - the node type.

---

``` public class PathFinder<N> extends java.lang.Object ```

This class is an implemenation of an algorithm for finding all pathways in a
directed graph.

**Author:**
:   This class was written and documented by Jeremiah Wright while in the
    Wagner lab.

---

**Constructor Summary**

`PathFinder(DirectedGraph<N> graph)`
  
          Constructs a new path finder for the provided graph.


| **Method Summary** | |
| --- | --- |
| `DirectedGraph<N>` | `getGraph()`             Returns the graph in which pathways will be computed. |
| `java.util.List<Path<N>>` | `getPaths()`             Returns a list of all paths. |
| `java.util.List<Path<N>>` | `getPaths(java.util.Collection<? extends N> sources, java.util.Collection<? extends N> sinks)`             Returns a list of all paths between the provided source and sink nodes. |
| `<T extends N,S extends N> java.util.List<Path<N>>` | `getPaths(T source, S sink)`             Returns a list of all paths between the provided source and sink nodes. |
| `int` | `paths()`             Returns the total number of paths. |
| `int` | `paths(java.util.Collection<? extends N> sources, java.util.Collection<? extends N> sinks)`             Returns the number of paths between the provided source and sink nodes. |
| `<T extends N,S extends N> int` | `paths(T source, S sink)`             Returns the number of paths between the provided source and sink nodes. |

**Methods inherited from class java.lang.Object**

`clone, equals, finalize, getClass, hashCode, notify, notifyAll, toString, wait, wait, wait`

**Constructor Detail**

### PathFinder

```
public PathFinder(DirectedGraph<N> graph)
```

:   Constructs a new path finder for the provided graph.

    **Parameters:**: `graph` - the directed graph in which pathways will be computed.


**Method Detail**

### getGraph

```
public DirectedGraph<N> getGraph()
```

:   Returns the graph in which pathways will be computed.

    :   **Returns:**: the graph in which pathways will be computed.

---


### getPaths

```
public java.util.List<Path<N>> getPaths()
```

:   Returns a list of all paths.

    :   **Returns:**: a list of all paths.

---


### getPaths

```
public java.util.List<Path<N>> getPaths(java.util.Collection<? extends N> sources,
                                        java.util.Collection<? extends N> sinks)
```

:   Returns a list of all paths between the provided source and sink nodes.

    :   **Parameters:**: `sources` - the collection of nodes at which all returned paths will begin.: `sinks` - the collection of nodes at which all returned paths will end. **Returns:**: a list of all paths between the provided source and sink nodes.

---


### getPaths

```
public <T extends N,S extends N> java.util.List<Path<N>> getPaths(T source,
                                                                  S sink)
```

:   Returns a list of all paths between the provided source and sink nodes.
    Each path in the returned list will begin at the source node and end at
    the sink node.

    :   **Type Parameters:**: `T` - a type extending the primary node type.: `S` - a type extending the primary node type. **Parameters:**: `source` - the node at which all returned paths will begin.: `sink` - the node at which all returned paths will end. **Returns:**: a list of all paths between the provided source and sink nodes.

---


### paths

```
public <T extends N,S extends N> int paths(T source,
                                           S sink)
```

:   Returns the number of paths between the provided source and sink nodes.

    :   **Type Parameters:**: `T` - a type extending the primary node type.: `S` - a type extending the primary node type. **Parameters:**: `source` - the node at which all computed paths will begin.: `sink` - the node at which all computed paths will end. **Returns:**: the number of paths between the provided source and sink nodes.

---


### paths

```
public int paths()
```

:   Returns the total number of paths.

    :   **Returns:**: the total number of paths.

---


### paths

```
public int paths(java.util.Collection<? extends N> sources,
                 java.util.Collection<? extends N> sinks)
```

:   Returns the number of paths between the provided source and sink nodes.

    :   **Parameters:**: `sources` - the collection of nodes at which all computed paths will begin.: `sinks` - the collection of nodes at which all computed paths will end. **Returns:**: the number of paths between the provided source and sink nodes.


---


|  |  |  |
| --- | --- | --- |
| **Overview** \| **Package** \| **Class** \| **Use** \| **Tree** \| **Deprecated** \| **Index** \| **Help** | |  |
| PREV CLASS   NEXT CLASS | **FRAMES**    **NO FRAMES**     **All Classes** |  |
| SUMMARY: NESTED \| FIELD \| CONSTR \| METHOD | DETAIL: FIELD \| CONSTR \| METHOD |  |


---
